# Formulation of Garlic Essential Oil-assisted Silver Nanoparticles and Mechanistic Evaluation of their Antimicrobial Activity against a Spectrum of Pathogenic Microorganisms

**DOI:** 10.2174/0115680266322180240712055727

**Published:** 2024-07-31

**Authors:** Ashirbad Sarangi, Bhabani Shankar Das, Lipsa Leena Panigrahi, Manoranjan Arakha, Debapriya Bhattacharya

**Affiliations:** 1 Centre for Biotechnology, Siksha ‘O’ Anusandhan (Deemed to be University), Bhubaneswar, Odisha, 751003, India;; 2 Department of Biological Sciences, Indian Institute of Science Education and Research (IISER) Bhopal, Bhopal, India

**Keywords:** Green synthesis, Garlic essential oil, Silver nanoparticles, Antimicrobial activity, Antibiofilm activity, Reactive oxygen species

## Abstract

**Background:**

The synthesis of nanoparticles using the principle of green chemistry has achieved huge potential in nanomedicine. Here, we report the synthesis of silver nanoparticles (AgNPs) employing garlic essential oil (GEO) due to wide applications of GEO in the biomedical and pharmaceutical industry.

**Objective:**

This study aimed to synthesise garlic essential oil-assisted silver nanoparticles and present their antimicrobial and antibiofilm activities with mechanistic assessment.

**Methods:**

Initially, the formulation of AgNPs was confirmed using different optical techniques, such as XRD, FT-IR, DLS, zeta potential, SEM, and EDX analysis, which confirmed the formulation of well-dispersed, stable, and spherical AgNPs. The antimicrobial and antibiofilm activity of GEO-assisted AgNPs was evaluated against a spectrum of pathogenic microorganisms, such as Gram-positive (*S. aureus* and *B. subtilis*) and Gram-negative (*E. coli* and *P. aeruginosa*) bacteria.

**Results:**

The AgNPs exhibited remarkable antimicrobial and anti-biofilm activity against all tested strains. The mechanism behind the antimicrobial activity of AgNPs was explored by estimating the amount of reactive oxygen species (ROS) generated due to the interaction of AgNP with bacterial cells and observing the morphological changes of bacteria upon AgNP interaction.

**Conclusion:**

The findings of this study concluded that ROS generation due to the interaction of AgNPs with bacterial cells put stress on bacterial membranes, altering the morphology of bacteria, exhibiting remarkable antimicrobial activity, and preventing biofilm formation.

## 1 INTRODUCTION

Essential oils have been used for centuries in medicine and aromatherapy treatments [1]. They are volatile, lipid-soluble liquids with transparent properties with different beneficial effects, such as bactericidal, fungicidal, insecticidal, virucidal, anti-parasitical, anti-inflammatory, anti-carcinogenic, and anti-diabetic remedies [2, 3]. These properties of essential oils make them substantially relevant for a wide range of uses, such as in the food and pharmaceutical industries [4]. Furthermore, garlic (*Allium sativum* L.) belongs to the Liliaceae family and possesses essential oils with remarkable properties. Garlic essential oil (GEO) is characterized by its predominant constituents, including Diallyl sulfide (DS), Allyl methyl disulfide (AMDS), Allyl methyl trisulfide (AMTS), Diallyl disulfide (DDS), and Diallyl trisulfide (DTS), and demonstrates significant antimicrobial potential with food preservation and safety [5]. Its discernible antibacterial efficacy against microorganisms like *Cronobacter sakazakii*, *Staphylococcus aureus,* and other bacteria has been shown to have notable effects [6, 7]. However, the relevant use of GEO is still restricted due to its high volatility, water insolubility, low bioavailability, and low physicochemical instability, which prevent its use in antibacterial effectiveness and food preservation [8]. To address these constraints, a green synthesis method using metallic nanoparticles can be an alternative way to enhance its utility with its advantageous efficacy. These days, the production of nanomaterials through essential oils is in demand since it may offer a viable substitute for metallic nanoparticles, helping to boost their stability and bioavailability.

The utility of nanoparticles in this context is emphasised by their size properties, which allow us to manipulate, characterise, and fabricate materials [9, 10]. In addition, the trend of environmentally friendly silver nanoparticle (AgNP) synthesis leverages safe reaction processes and considerable antibacterial potential. This heightened antimicrobial potential of AgNPs is attributed to their extensive surface area, which allows them to interact with microorganisms and inhibit their growth at low concentrations [9, 11]. It also makes them valuable for controlling microorganism proliferation in the food industry and ESKAPE pathogens [11]. The WHO (World Health Organization) has identified ESKAPE pathogens as the most concerning community-spreading pathogens. Out of our test pathogens, *Staphylococcus aureus*, *Pseudomonas aeruginosa*, and *Escherichia coli* are highly variable strains and cause extreme coinfection with virulent activity and antibiotic-resistant infections.

Moreover, they pose a public health threat, causing infections, such as bloodstream infections, pneumonia, wound infections, and urinary tract infections. The increasing resistance to routinely used antibiotics distinguishes ESKAPE infections from other diseases. This growing resistance, combined with the therapeutic importance of these bacteria in the medical profession, necessitates understanding their resistance mechanisms and the development of novel antibiotics to tackle them. Common resistance mechanisms include the development of enzymes that damage the structure of antibiotics, alteration of the antibiotic's target site so that it no longer binds well, efflux pumps, and biofilm production [12].

Despite different plant extracts being used for AgNP synthesis, ethnomedicinal plant essential oils still need to be explored in the green synthesis of AgNPs [13]. While essential oil from garlic has been extensively studied for its antimicrobial activity against different pathogens, till now, no prior research has been investigated using silver nanoparticles synthesised from GEO against some Gram-positive and -negative microorganisms [14-16]. Furthermore, the utilization of nanomaterials using essential oil is currently in its nascent phase. To our knowledge, there is currently no published data on the use of nanoparticles derived from GEO. Despite this, there is an increasing interest in exploring the utilisation of GEO-synthesised nanoparticles as a novel approach to enhance the efficacy. It is the first report about GEO nano-form, which can be used as a nanoparticle with its effective bioactivity and can be used for remediation and industrial purposes in alternate. Therefore, our study endeavours to examine the potential of GEO-synthesised silver nanoparticles (AgNPs) using a sustainable, eco-friendly method. These synthesised silver nanoparticles were further characterized using FTIR, XRD, DLS, Zeta potential, EDX, and Scanning Electron Microscopy (FE-SEM) techniques. Additionally, the antimicrobial and antibiofilm efficacy of GEO-assisted nanoparticles was evaluated against four pathogens, including *S. aureus, B. subtilis, E. coli*, and *P. aeruginosa,* with GEO and silver nitrate (AgNO_3_) as controls.

Additionally, an ROS detection assay was carried out to find out the probable mechanism of action of nanoparticles. The implications of this study will introduce a novel strategy for utilising GEO as a plant-based antimicrobial additive agent against foodborne and ESKAPE pathogens. However, findings from this experiment could be implicated in the food industry and healthcare settings.

## 2 MATERIALS AND METHODS

### 2.1 Bacterial Strains and Chemicals

*Staphylococcus aureus* (MTCC 96), *Bacillus subtilis* (MTCC 441), *Escherichia coli* (MTCC 443), and *Pseudomonus aeruginosa* (MTCC 2488) were purchased from the Institute of Microbial Technology India and were cultured in nutrient broth (Cat. No. M002-500G) and nutrient agar (Cat. No. M001-500G). For further experiments, bacteria were transferred to Mueller Hinton Broth (Cat. No. M391-500G) and Mueller Hinton Agar (Cat. No. M173-500G). Silver nitrate was purchased from Sigma Aldrich (Cat no-204390). 2′,7′ -Dichlorodihydrouorescein diacetate (DCFH-DA Cat no-4091-99-0) was obtained from Cayman chemicals, USA.

### 2.2 Extraction of Essential Oil from Garlic Bulbs

The Indian Garlic (*Allium sativum* L.) cloves were collected from the local market of Bhubaneswar, India. Approximately 300 g of fresh garlic bulbs were peeled out and chopped with distilled water using a blender for hydro-distillation through boiling and maintained at ambient temperature (20 °C) for 5h using a Clevenger unit (European Pharmacopoeia). The yield percentage of extracted GEO was 0.17% (V/W), stored at -20 °C for further analysis.

### 2.3 Green Synthesis of GEO-assisted AgNPs

GEO was initially diluted with Acetone (1:170), with the aim of properly mixing it into the medium. About 200 mL solution of 10 mM silver nitrate was prepared, and pH was adjusted to 8. The diluted essential oil was added to the solution and vigorously stirred for 24 hours. A physical change of colour was observed, which is yellowish brown, indicating the synthesis of silver nanoparticles from GEO. Nanoparticles were washed with distilled water to remove the extra oil content and dried using a hot air oven. Dried nanoparticles were used for further analysis.

### 2.4 Characterisation of AgNPs

The XRD analysis of AgNPs was performed using the smart lab version of the Rigaku X-ray Diffractometer (Tokyo, Japan), using Cu-Kα radiation, at a scan rate of 20°/min with a step size of 0.05° over a 2θ range of 20 to 80. To study the different phases of synthesised nanoparticles, X’-pert high score software with a search and match facility was used. To analyse surface morphology and size, different characteristics techniques, such as FE-SEM with EDX (SEM, Jeol-JSM-IT800, USA), were used in this study. The surface potential and hydrodynamic size analysis of the biosynthesised AgNPs were done using DLS (Dynamic light scattering) and a zeta analyser (Malvern Zetasizer Nano ZS90, Netherlands). The functional group detection was conducted by FTIR spectrometer (Jasco-4200, USA), where the scanning range was kept from 4000 to 400 cm^-1^.

### 2.5 Antibacterial Activity of GEO-assisted AgNPs

#### 2.5.1 Evaluation of Minimum Inhibitory Concentration (MIC) and Minimum Bactericidal Concentration (MBC)

MIC was determined by micro broth dilution method following the Clinical and Laboratory Standards Institute (CLSI) [17, 18] guidelines, using Mueller Hinton Broth as liquid media. Overnight culture bacteria at 0.6 O.D. with 10^4^ cells/ mL were plated on a 96-well plate with different concentrations of GEO, AgNPs, and silver nitrate (500, 250, 100, 50, 25 µg/mL) for 14 hours at 37°C. The plates were observed after overnight incubation, and the MIC dose indicated no visible growth. 1x Alamar blue (Invitrogen) was added per well, and a change in colour from blue (oxidise form) to pink (reduced form) was recorded after 1 h of incubation. The MIC was determined at the concentration with a low dose having no change in colour. About 2 µL of each concentration was plated on MHB agar, and the plates were incubated overnight to determine the MBC. The experiments were done in triplicates.

#### 2.5.2 Growth Inhibitory Effect of AgNPs

Based on the MIC, the growth kinetic study of nanoparticles was examined. The mid-log phase culture of bacteria (10^4^ cells/well) was treated with different concentrations of nanoparticles. Optical density at 600 nm was checked for 24 hours in a periodic interval. Data were recorded, and the graph was plotted.

#### 2.5.3 Estimation of ROS Generation Upon AgNP Interaction with Bacterial Cells

2′,7′-dichlorodihydro-fluorescein diacetate (DCFH-DA) was used to measure the amount of ROS that was produced by AgNPs. Test organisms included both Gram-positive (*S. aureus* and *B. subtilis*) and Gram-negative (*E. coli* and *P. aeruginosa*) bacteria. Overnight culture bacteria were diluted with LB broth with 10^4^ cells per well, and AgNPs were treated with MBC, MIC, half MIC, and quarter MIC doses. Afterwards, overnight incubation of 5µL of 10 mM 2′,7′-dichlorodihydro-fluorescein diacetate was put into each well, and the fluorescence was checked at 503 nm excitation and 523 nm emission.

### 2.6 Antibiofilm Activity of GEO-assisted AgNPs

Focusing on antibacterial activity, the antibiofilm activity was evaluated against all the tested strains. The overnight culture of bacteria was treated with different concentrations of silver nitrate and AgNPs in sterile test tubes containing LB (Luria-Bertani Broth) with 2% glucose. After visible biofilm formation, the tubes were washed thrice with 1X PBS (phosphate buffer saline) and then stained with 0.1% crystal violet (*S. aureus* and *B. subtilis*) and safranine (*E. coli* and *P. aeruginosa)* for 10 minutes and was added with 33% glacial acetic acid. Optical density was checked at 570 nm for crystal violet and 492 nm for safranine.

### 2.7 Estimation of Extracellular Polysaccharide Content of Biofilm

EPS was extracted from the biofilm of tested organisms with minor modifications [19]. After the biofilm formation of the tested strains, the total biofilm content was taken out to individual tubes and centrifuged at 6000 rpm, and then supernatant was taken out and stored. About 10 mM of EDTA was added to each tube containing the residual pellet and then again centrifuged at 6000 rpm. The supernatant was collected and mixed with the previous one. About 2.2 volumes of chilled ethanol were added to the total supernatant collected and then set to incubate for 1 hour at -20°c. Then, the mixture was centrifuged, and the supernatant was discarded. Distilled water was added to the pellet and set to dissolve. After extraction, polysaccharide content was estimated using the phenol sulphuric acid method [20].

### 2.8 Scanning Electron Microscopy of Biofilm

For SEM analysis, adherent biofilms on coverslips were washed with distilled water first and then with 1X PBS. About 2.5% glutaraldehyde was added to the coverslips for fixation. Coverslips were subjected to incubation for 20 min at 4°C. After incubation, coverslips were washed with 1X PBS and then dried with varying concentrations of absolute alcohol and then air dried at room temperature. Subsequently, after drying, coverslips were stored at 4°C overnight and then subjected to SEM imaging.

### 2.9 MTT Assay

HEK 293 cells were cultured in a DMEM growth medium supplemented with 7.5% FBS. Cells were maintained at 37 °C in a 5% CO2 atmosphere and passed by trypsinization every 3-4 days to maintain subconfluence. 5x10^3^ cells were seeded in each well of 96 well plates for MTT assay. The following day, the cells were treated with an 800 μg/mL dose of the given nanoparticles. After 24 hours of treatment, an MTT assay was carried out using standard protocol to check the percentage of cell viability. Briefly, 20 µL of MTT was added to each well, and the plate was incubated for 3 hours at 37°C. Following incubation, absorbance was taken at 570nm in an ELISA plate reader.

### 2.10 Statistical Analysis

The data obtained from the experiments were analysed using Minitab statistical software for Windows. To determine the significance, a one-way analysis of variance (ANOVA) was conducted, followed by Dunnett's post hoc test. A p-value of ≤ 0.05 was considered to indicate significance. The results were presented as the mean ± standard deviation (SD)/standard error of the mean (SEM) of three biological experiments for each treatment, with three appropriate replicates conducted under similar conditions unless otherwise specified.

## 3 RESULTS AND DISCUSSION

Garlic is a majorly cultivated bulb crop worldwide because of its extensive use in food and medicine [21, 22]. By utilizing essential oils as reducing and stabilizing agents, nanoparticles like AgNPs with unique physicochemical properties can be fabricated, making them suitable for a wide range of applications [23-26]. Biosynthesized AgNPs from GEO, in this study, have been used to investigate antimicrobial and antibiofilm properties.

### 3.1 Assessment of Yield and Composition of Essential Oil Extracted from Garlic

The garlic bulbs essential oil of *Allium sativum* displayed a pale yellow colour and emitted a robust aroma essence. Hydro-distillation, using a Clevenger-type apparatus, obtained an average essential oil yield (GEO) percentage of 0.17% (v/w) based on the dry weight. Subsequently, in line with our esteemed previous report, a detailed analysis was conducted to identify the major volatile constituents using gas chromatography-mass spectrometry (GC-MS) [27]. The study revealed that DTS and DDS collectively represented 62.9% of the total oil composition, indicating a significant presence of sulfur components within the essential oil. This observation underscores the potential importance of DTS and DDS in contributing to the overall characteristics of the garlic essential oil. Furthermore, these findings also indicate the intriguing prospect of further exploration into the bioactive properties of the essential oil and signify a promising avenue for deeper investigations into its pharmacological and medicinal potential.

### 3.2 Optical Characterization of GEO-assisted AgNPs

#### 3.2.1 Crystallinity Analysis of Synthesised AgNPs

**Fig. (1):**
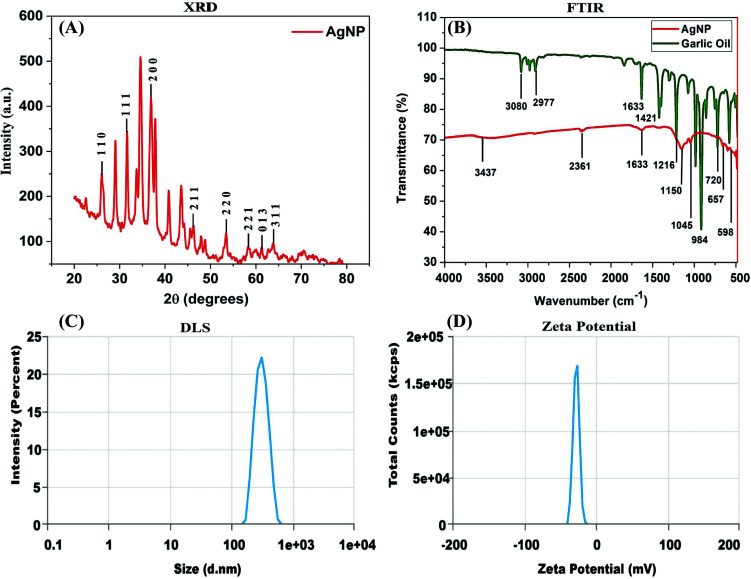
Characterisation for biosynthesised AgNPs using garlic essential oil. (**A**) Represents formation of AgNPs confirmed through X-ray Diffraction analysis (**B**) FTIR spectroscopy analysis for identification of biomolecules present in garlic essential oil as, with green colour indicates garlic essential oil and red colour indicates biosynthesised AgNPs (**C**) DLS analysis of AgNPs (**D**) Zeta potential of newly synthesised AgNPs.

The crystalline characteristics of synthesized AgNPs were investigated using an X-ray diffractometer (XRD) analysis. The X-ray diffraction pattern of AgNPs, displayed a face-centred cubic structure with distinct four diffraction planes 111, 200, 220, and 311, corresponding to 2θ angles of 37.934°, 44.142°, 64.678°, and 77.549° respectively, (Fig. **1A**). These diffraction patterns also match well with standard JCPDS card number 00-001-1164, confirming the face-cantered cubic structure with space group and space group number Fm3m, 225 respectively [28]. Furthermore, the peaks in the spectral data are strong and narrow, demonstrating the crystalline nature of AgNPs. Also, the size of the AgNPs was determined by using the Debyes Scherrer equation: D = (kλ/β cos θ). Where D is particle size; K is Schrerrer’s constant (0.9); λ is the wavelength of X-ray (1.540×10^−10^ m) used for performing XRD; β is full width at half maximum (FWHM), and θ is Bragg diffraction angle [29]. Overall, the XRD analysis indicates the crystalline nature of the AgNPS, with well-defined crystal planes contributing to their structural integrity and properties.

#### 3.2.2 Bond Level Characterization of Biosynthesized AgNPs

The Fourier-Transform Infrared (FTIR) spectrum of biosynthesized AgNPs exhibits distinctive peaks that provide valuable insights into their chemical composition and surface properties. One of the prominent features in the FTIR spectrum of silver nanoparticles is typically observed in the region of 400-600 cm^-1^, known as the “metallic bonding” region [30]. A strong absorption peak at specific wavenumbers further elucidated the molecular composition of the AgNPs. The peaks obtained at 1633 represent the carbonyl and carboxylic (C=O) group and stretching bands of peptide linkage. The peak at 3080 cm^−1^ corresponds to the asymmetric stretching vibration of =CH_2_. The peak at 2977 cm^−1^ is attributed to the symmetric stretching vibration of C-H. The peaks at 1633 cm^−1^, 1421 cm^−1^, and 1216 cm^−1^ correspond to the stretching vibration of the C=C allyl group, OH deformation vibration, C-O stretching, and CH_2_=CH stretching, respectively. Similarly, the peak values at 984 cm^−1^ correspond to C-O stretching vibration (Fig. **1B**). Comparing our findings with those reported by Tavares *et al.* [30] for C-H, =CH_2_, C=O, C=C, C-O, and SPI groups, consistency was found in the results. Thus, the results indicated the presence of GEO organosulfur bioactive compounds incorporated within the nanoparticles. This combined analysis of the FTIR spectrum and comparison with existing research highlights the specific chemical groups and bonds present in the biosynthesized AgNPs, shedding light on their composition and potentially their biological activity.

#### 3.2.3 Hydrodynamic Size and Zeta Potential Analysis of Biosynthesised AgNPs

Shape and size are crucial criteria for determining nanoparticles used as catalysts, biosensors, and in medical applications. Previous research on the nanoparticles irregular form and size from biological sources, particularly plants, has been published [30-32]. In contrast, our synthesised AgNPs show a smaller size particle, which highlights the importance of our biosynthesised silver nanoparticle from GEO. To correlate our findings, DLS measures particle hydrodynamic size, which seems to be larger than that of core particles in most circumstances due to the presence of a hydration layer around the particles [33]. The hydrodynamics size of AgNPs was determined using DLS (Dynamic Light Scattering) spectroscopic technique. According to DLS measurements, the biogenic AgNPs ranged from 200-300 nm, with an average hydrodynamic size of approximately 284.9 nm. However, the majority of the particles were found to be around 200 nm in size (Fig. **1C**). Furthermore, the zeta potential (Z) value of AgNPs was determined and found to be -27.56 mV, and this magnitude of 'Z' reflected moderate particle stability (Fig. **1D**). This negative zeta potential value of the silver nanoparticles attributed to the bioorganic components present in the GEO, potentially acts as a protective layer. The strong negative values indicate electrostatic repulsion between the particles, facilitating the formation of stable silver nanoparticles that do not clump together.

#### 3.2.4 Morphological and Elemental Analysis of GEO-mediated AgNPs

**Fig. (2):**
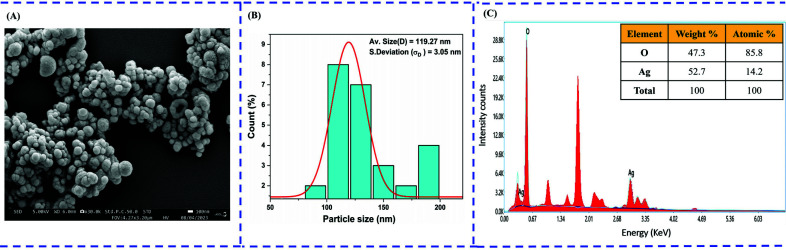
Morphological and elemental representation of biosynthesised AgNPs (**A**) SEM micrograph of synthesized AgNPs (**B**) Particle size distribution of biosynthesised AgNPs from garlic essential oil (**C**) EDX spectrum analysis of AgNPs.

Field Emission Scanning Electron Microscopy (FE-SEM) was used to examine the surface morphology of biosynthesized AgNPs. The images here depict the crystalline particles having spherical and regular morphology with an average size of 119.27 nm (Fig. **2A** and **B**). Energy-dispersive X-ray spectroscopy (EDX) was also employed to determine the elemental composition of biosynthesized AgNPs. As demonstrated in the EDX report, silver was detected at a weight % of around 52.7% with an atomic % of 14.2, confirming the synthesis of AgNPs from GEO (Fig. **2C**).

### 3.3 Antimicrobial Efficacy of GEO-mediated AgNPs

#### 3.3.1 Sensitivity of AgNPs on Gram-positive and Gram-negative Bacteria

**Fig. (3):**
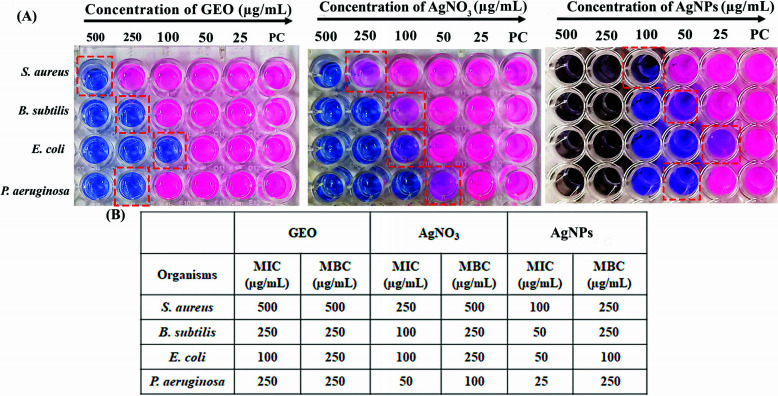
*In-vitro* antimicrobial activity of AgNPs against two Gram-positive and two Gram-negative microorganisms: *S. aureus, B. subtilis, E. coli,* and *P. aeruginosa*. (**A**) Represents MIC of GEO, AgNO_3,_ and AgNPs as determined by Alamar blue assay. (**B**) Summarizes antibacterial effectiveness of GEO, AgNO_3_, and AgNPs in terms of MIC and MBC.

First, we observed the antibacterial activity of GEO and silver nitrate as controls for our experiment. The MIC and MBC of GEO and AgNO_3_ against all tested strains are given in (Fig. **3A** and Figs. **S1** and **2**). The garlic oil-assisted AgNPs were initially tested in a dose-dependent antibacterial activity against two Gram-positive and two Gram-negative microorganisms. In contrast to the control, growth inhibitory activity was observed at a particular concentration under study. Although different AgNP concentrations are present, *P. aeruginosa* is the organism that works best at a dose of 25 µg/mL. *B. subtilis* and *E. coli* showed the same MIC at 50 µg/mL, followed by *S. aureus* at 100 µg/mL (Fig. **3A**). MBC was the same for all the tested strains at 250 µg/mL (Fig. **S3**). These findings suggest the potential of green-synthesised AgNPs as effective antibacterial agents, with susceptibility observed among the different bacterial species. A research was carried out by Momani *et al.*, to assess the antibacterial and antibiofilm efficacy of biosynthesized AgNPs against six biofilm-forming clinically isolated specimens of Pseudomonas. The findings showed MIC at 15.6 µg/mL and MBC at 31.25 µg/mL. The biosynthesized AgNPs showed significant inhibitory effects on growth, biofilm formation, and metabolism, as well as reduced quorum sensing in all strains (Fig. **3B**) [34]. Another work by Hamida *et al.* evaluated the inhibitory effect of biosynthesized silver nanoparticles against 5 pathogenic bacteria. The results showed that biosynthesized AgNPs targeted the virulence of MDR strains of *S. aureus* by apoptotic body formation and cell wall damage leading to bacterial cells [35]. Similarly, the antimicrobial propensity of AgNPs at a low concentration of *B. subtilis* was mediated by the release of Ag^+^ ions, as shown by Hsueh *et al.* [36]. Another work carried out by Li *et al.*, where *S. aureus* was exposed to 50 µg/mL of AgNPs, resulted in a condensed state of DNA and reduced ability of replication [37]. In addition to the above research, Campo-Baleno *et al.* also reported the antimicrobial effect of Ag nanoparticles against multi-drug *P. aeruginosa* [38]. Salem *et al.* also reported significant antimicrobial activity and excellent biocompatibility against *E. coli* U_12_ upon the addition of biologically synthesized silver nanoparticles [39].

#### 3.3.2 Growth Inhibitory Effect of AgNPs

Following the determination of MIC, a comprehensive assessment of growth kinetics study was performed against all tested strains to assess the antibacterial effectiveness of biosynthesized AgNPs. The growth kinetics were examined in 10^4^ cells of sensitive bacterial strains with their respective controls to evaluate the growth inhibitory property of GEO synthesized AgNPs in a dose-dependent manner (500, 250, 100, 50, 25 µg/mL). The optical density measurements at 600 nm over 24 hours revealed that the lower concentrations of 25 and 50 μg/mL showed significant bacterial growth, which was observed among all the strains. However, the bacterial growth was moderately inhibited, observed at 100 μg/mL, as evident from Fig. (**4**). Remarkably, the highest concentrations of 500 and 250 μg/mL exhibited complete restriction of bacterial growth, suggesting low viability of the cells (Fig. **S4**). Overall, the above findings highlight the antibacterial activity of the green-synthesized AgNPs, with higher concentrations demonstrating enhanced efficacy in inhibiting bacterial growth. Furthermore, the growth kinetics data provide valuable insights into the dynamic interactions between the AgNPs and bacterial cells, highlighting the potential of these nanoparticles as effective antimicrobial agents.

### 3.4 Antibacterial Effect of Synthesized AgNPs through ROS Generation Activity

In microorganisms, synthesized silver nanoparticles (AgNPs) have the potential to elicit nonviability through mechanisms that involve either augmentation of interfacial or intracellular reactive oxygen species (ROS) generation or the denaturation of cellular components upon interaction at the interface or a combination of both [40]. As illustrated in Fig. (**5**), the relative oxidation product of 2’,7’-dichlorofluorescein diacetate (DCFH-DA) serves as an equivalent representation of ROS levels produced in cultures of *S. aureus*, *B. subtilis*, *E. coli*, and *P. aeruginosa* when subjected to varying concentrations of synthesized GEO-AgNPs formulations, including minimum bactericidal concentration (MBC), minimum inhibitory concentration (MIC), half MIC, and quarter MIC concentrations. Consequently, this dye is commonly employed as an indicator for quantifying the relative ROS levels. Notably, less ROS production was observed in untreated bacteria, as depicted in Fig. (**5**), indicating a baseline ROS generation under non-stress conditions.

However, the penetration of AgNPs during the mid-log phase of growth resulted in a substantial increase in ROS generation by 4-fold between 16 hours and 20 hours, signifying a remarkable augmentation capable of inducing oxidative stress in selective strains. Furthermore, the production of ROS in four selected bacterial species was found to be dose-dependent, with a comparatively higher ROS generation observed in *P. aeruginosa* at concentrations of 250 µg/mL (MBC) and 25 µg/mL (MIC), followed by *E. coli* at 250 µg/mL (MBC) and 50 µg/mL (MIC). *S. aureus* at 250 µg/mL (MBC) and 100 µg/mL (MIC) and *B. subtilis* at 250 µg/mL (MBC) and 50 µg/mL (MIC) also showed high ROS production. Half and quarter MIC showed moderated ROS production in all the tested strains at 20 hours, followed by 16 hours. In contrast, the possible mechanism underlay that when silver ions are treated to bacterial strains, the reactive oxygen species (ROS) and cellular oxidative stress are produced within the bacteria through the interaction of silver ions, and disulfide bonds of the enzymes are in charge of cellular metabolism and thiol groups, especially respiratory enzymes are damaged by ROS because they break the double bonds that hold fatty acids in the membrane generating more free radicals. The interaction of metal ions with thiol and sulfhydryl groups in the bacterial cell membrane protein can be linked to the antibacterial activity of AgNPs [25]. This process may decrease the bacterial cell's permeability and cellular respiration and ultimately cause cell death [41]. However, Ag can also bind to bacterial DNA, denaturing it and preventing replication. Additionally, AgNPs have many silver atoms; they can operate as an antibacterial agent against both Gram-positive and -negative bacteria by entering the pathogens' cells and preventing DNA gyrase from working [42]. An overview of our obtained ROS results suggests that the underlying mechanism of AgNPs acts as an efficient antibacterial representative against our experimental strains.

### 3.5 Antibiofilm Activity of GEO-assisted AgNPs

On non-living surfaces, several bacteria retain the capacity of biofilms out of their own matrix composed of polymers, which makes it more difficult to get away from planktonic colonies [43]. The antibiofilm activity of AgNPs was evaluated against biofilm-forming *S. aureus*, *B. subtilis*, *E. coli*, and *P. aeruginosa*. Using these organisms’ anti-biofilm activity, green synthesized AgNPs were synthesised using the liquid antibiofilm method. From the Fig. (**6**), it can be observed that GEO-assisted AgNPs inhibited the total biofilm formation (MBIC: Minimum Biofilm Inhibition Concentration) for both *S. aureus* and *P. aeruginosa* at the same concentration of 200 µg/mL. *B. subtilis* biofilm was inhibited at 400 µg/mL followed by *E. coli* at 800 µg/mL. We also evaluated the antibiofilm activity of GEO and silver nitrate and found that both GEO and silver nitrate inhibited *P. aeruginosa* at 250 µg/mL and 50 µg/mL, respectively. *B. subtilis* and *E. coli* were inhibited by GEO at 250 µg/mL and 100 µg/mL, whereas silver nitrate inhibited *B. subtilis* and *E. coli* at 100 µg/mL. The highest inhibition was observed against *S. aureus* at 500 µg/mL for GEO and 250 µg/mL for silver nitrate (Figs. **S5** and **6**).

**Fig. (7):**
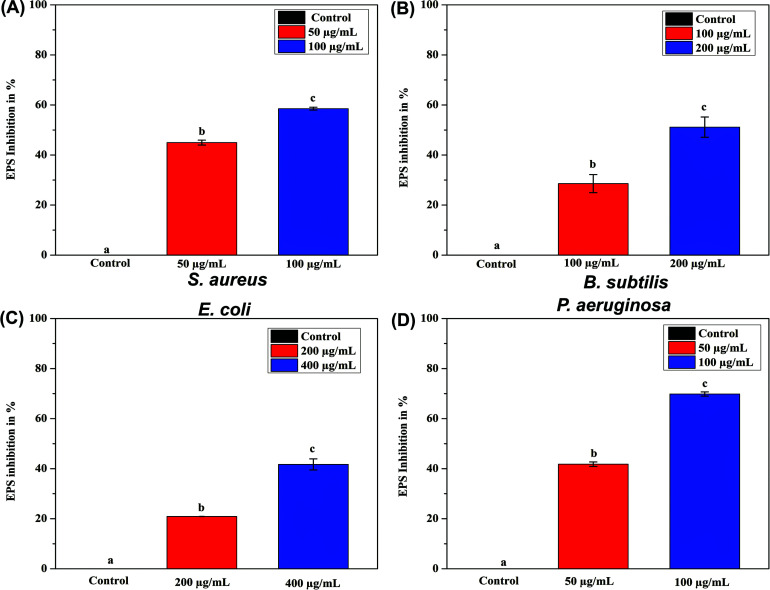
Qualitative analysis of extracellular polysaccharide production by biofilm-forming microorganisms upon treatment with AgNPs at sub-MBIC doses. The test microorganisms were (**A**) *S. aureus,* (**B**) *B. subtilis,* (**C**) *E. coli,* and (**D**) *P. aeruginosa*. The results are shown as mean ± SD of three independent experiments. Each alphabetical letter represents statistically significant different AgNPs treated groups and control group (without AgNPs), with p-value ≤ 0.05.

In previewing the above biofilm activity, biofilm has a closed structure of planktonic cells embedded with a high amount of extracellular polymeric substances (EPS) [44]. The percentage of EPS inhibition in the biofilms of the tested strains was quantified in a dose-dependent manner. We found a strong relationship between AgNPs' antibiofilm action and EPS concentration. We observed EPS from the biofilm of tested strains treated with various sub-MBIC dosages of AgNPs decline dose-dependently. The percentage of EPS inhibition was found to be high against *P. aeruginosa* at a dose of 100 µg/mL, showing 68%, followed by *S. aureus* at 58% at a dose of 100 µg/mL and *B. subtilis* at 51% at a dose of 200 µg/mL. The lowest percentage of inhibition was found against *E. coli* at 41% at a dose of 400 µg/mL (Fig. **7**).

**Fig. (8):**
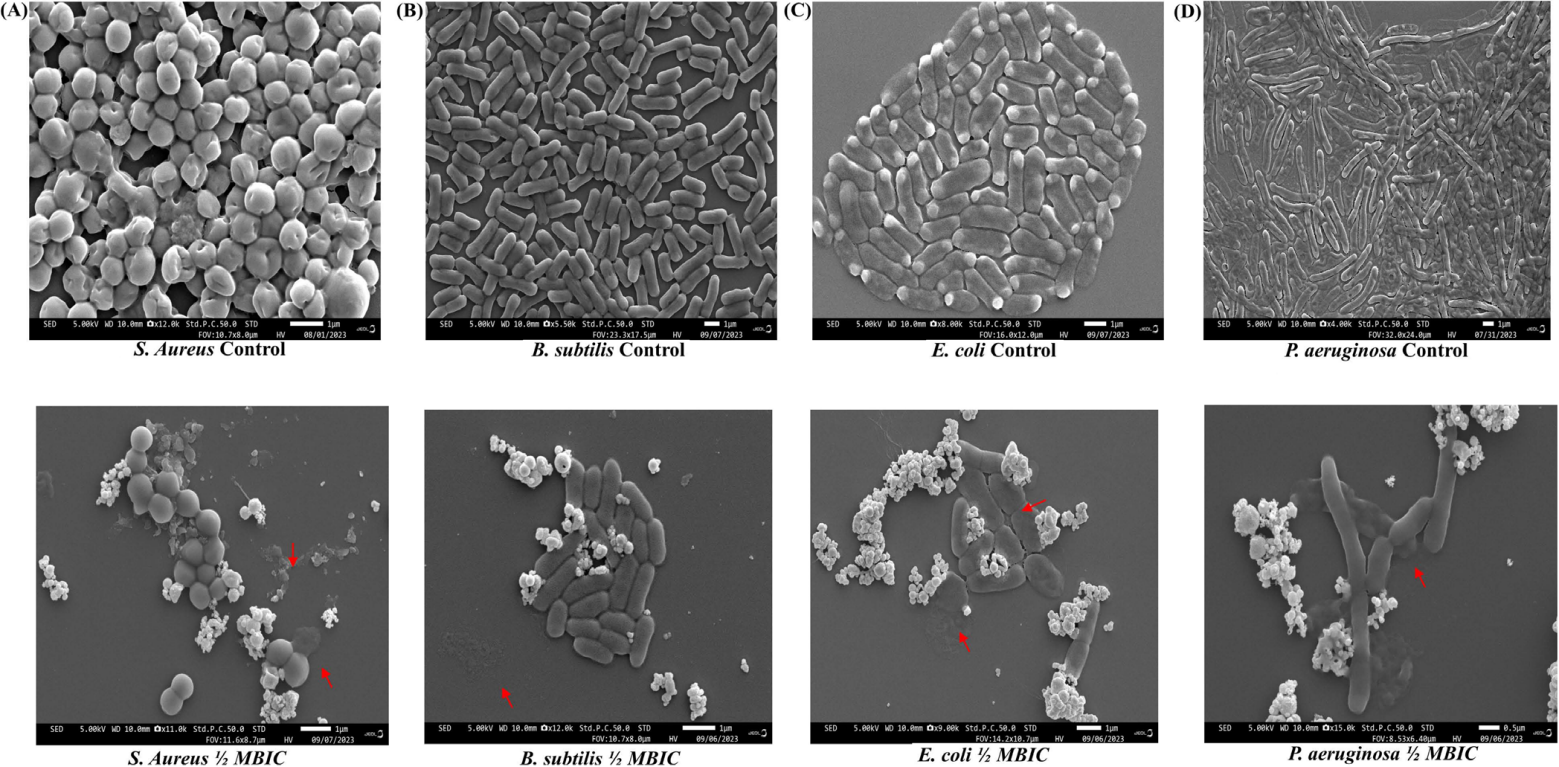
Scanning electron microscopy (SEM) images of biofilms of (**A**) *S. aureus*, (**B**) *B. subtilis*, (**C**) *E. coli*, and (**D**) *P. aeruginosa* treated with half of their minimum biofilm eradication concentration (MBIC) of silver nanoparticles (AgNPs) showed a reduction in biofilm biomass compared to control biofilms. Red arrows indicate the effects of AgNPs, such as cell shrinkage, rupture, nanoparticle uptake, and reduced biofilm populations. The scale bar represents 1µm.

Additionally, the spatial organization of the bacterial biofilm was determined using a scanning electron microscope (SEM). The SEM images clearly showed that the biomass present in the half MBIC dose was lower than the control (Fig. **8**). Some bacterial cells of all the tested strains were found to be damaged because of the uptake of AgNPs, which clearly indicates that AgNPs synthesized from GEO damages bacterial cells which does not allow the microorganism to form biofilm. Furthermore, SEM images of the cells after exposure to AgNPs showed morphological modifications and lyses of the outer membrane integrity. This effect might be an electrostatic response between AgNPs and bacteria. Thus, the study highlights the effectiveness of green synthesized AgNPs in combating biofilm formation by targeting EPS and disrupting bacterial cell integrity. These findings shed light on the potential of AgNPs as a promising antibiofilm agent against pathogenic bacteria, offering insights into novel strategies for biofilm control in non-living surfaces.

### 3.6 Cell Viability Check

**Fig. (9):**
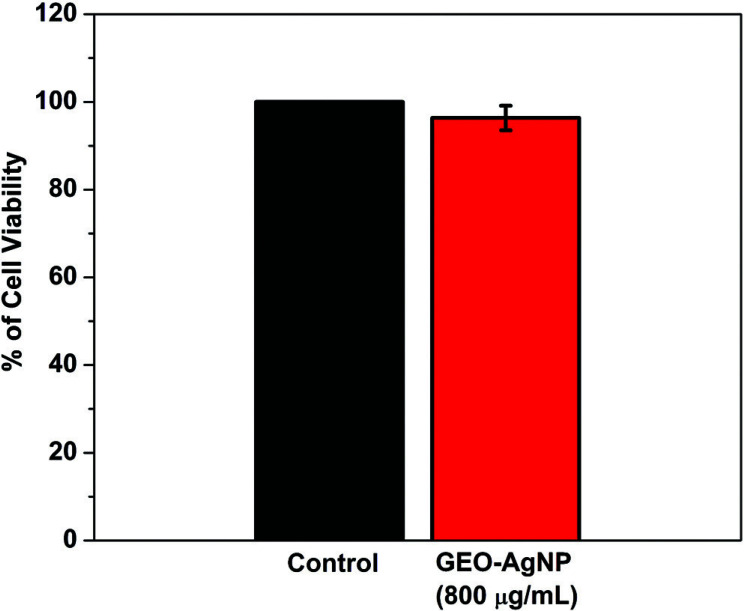
Percentage of cell viability by treatment of GEO-assisted AgNPs.

Following treatment of the cells with the nanoparticles for 24 hours, it was observed that the cell viability was unaltered with 800 μg/ml of GEO-assisted AgNPs treatment. Results showed that with 800 μg/ml, 95% of cells were viable compared to normal untreated control (Fig. **9**).

## CONCLUSION

The present study demonstrates a rapid and cost-effective approach for the synthesis of AgNPs using garlic essential oil as a proficient reducing agent. The formulation of AgNPs was confirmed through a compressive biophysical technique, such as X-ray Diffraction, FT-IR spectroscopy, dynamic light scattering, zeta potential, and FE-SEM-EDX analysis. FTIR spectroscopic analysis confirmed that the sulfur molecules present within the essential oil play a pivotal role in the reduction of silver ions to silver nanoparticles. The resulting synthesized AgNPs exhibit remarkable antibacterial activity against different foodborne pathogens, including *S. aureus*, *B. subtilis*, *E. coli*, and *P. aeruginosa*. Additionally, the findings of the study also demonstrated the potential of AgNPs *via* GEO as a potent anti-biofilm agent against the tested strains examined. Insight into the bactericidal mechanism of AgNP findings showed that garlic essential oil-mediated AgNPs could be used as a promising bactericidal agent to overcome antimicrobial resistance and prevent microbial contamination.

## Figures and Tables

**Fig. (4):**
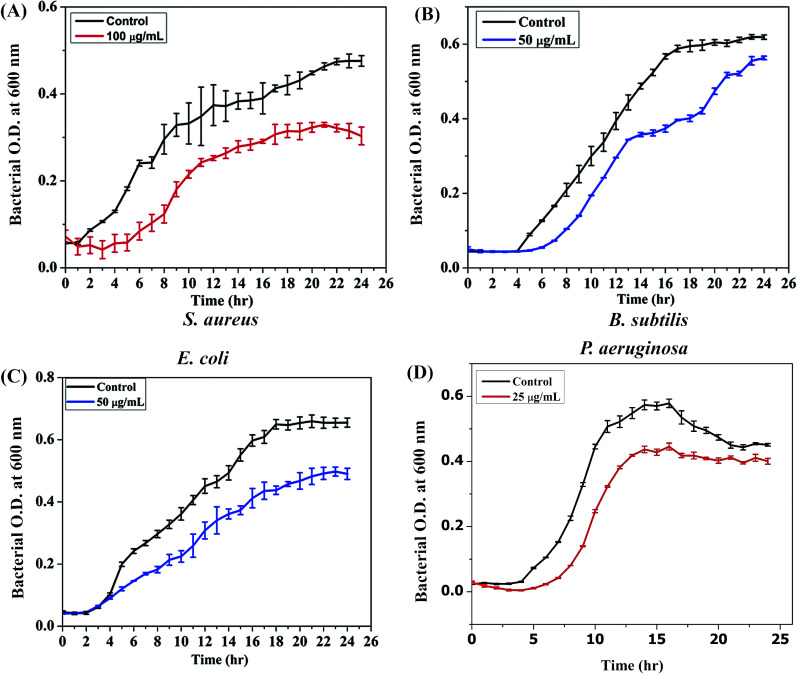
Illustrates MIC dose-dependent growth inhibitory effect of AgNPs in all tested strains (**A**) *S. aureus* (**B**) *B. subtilis* (**C**) *E. coli* (**D**) *P. aeruginosa.* The resulted data are presented as mean ± SD of three separate experiments.

**Fig. (5):**
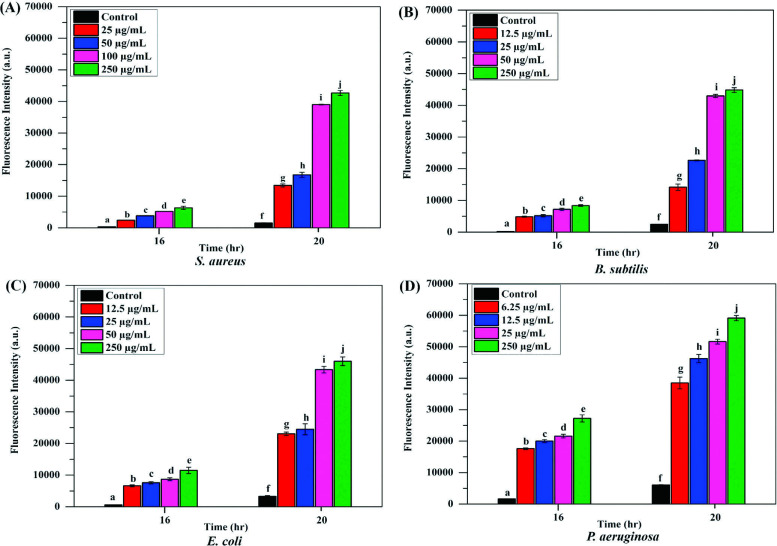
Silver nanoparticles (AgNPs) induce intracellular reactive oxygen species (ROS) generation in four tested bacteria: (**A**) *S. aureus*, (**B**) *B. subtilis*, (**C**) *E. coli*, and (**D**) *P. aeruginosa* at different concentrations, including MBC, MIC, half MIC, and quarter MIC. The results are shown as mean ± SD of three independent experiments. Each alphabetical letter represents a statistically significant difference between AgNPs-treated and the control group (without AgNPs), with a p-value ≤ 0.05.

**Fig. (6):**
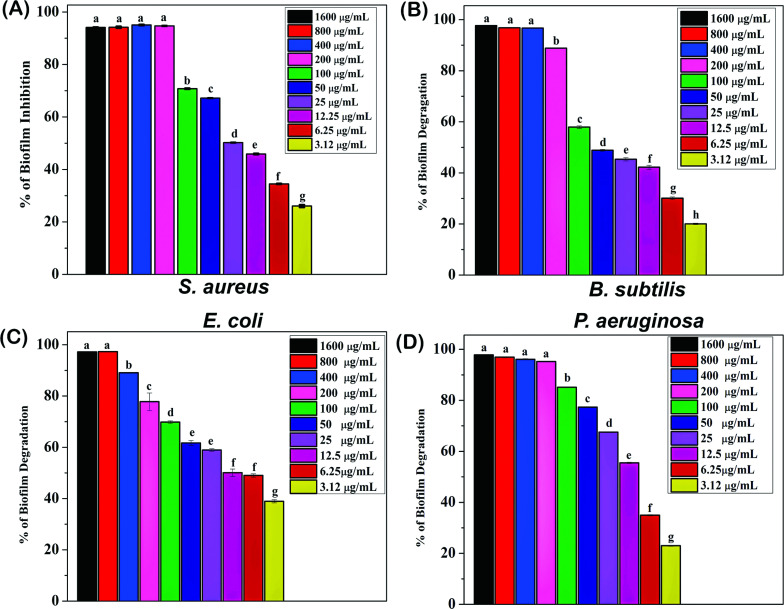
Antibiofilm activity of AgNPs against biofilm-forming microorganisms: (**A**) *S. aureus* (**B**) *B. subtilis* (**C**) *E. coli* (**D**) *P. aeruginosa.* The results are shown as mean ± SD of three individual biological experiments. Individual letters represent significant differences compared to the control group (without AgNPs) with a p-value ≤ 0.05.

## Data Availability

All the data and supporting information are provided within the article.
