# Supplementary material for: Formulation of Garlic Essential Oil-assisted Silver Nanoparticles and Mechanistic Evaluation of their Antimicrobial Activity against a Spectrum of Pathogenic Microorganisms
Source: Curr Top Med Chem. 2024 Jul 31;24(22):2000–12. doi: 10.2174/0115680266322180240712055727 (PMC11497146; doi:10.2174/0115680266322180240712055727)
Supplement: Supplementary file 1 [file CTMC-24-2000_SD1.pdf]

Supplementary Material

Formulation of Garlic Essential Oil-assisted Silver Nanoparticles and Mechanistic Evaluation of their Antimicrobial Activity Against a Spectrum of Pathogenic Microorganisms

Ashirbad Sarangi[1,#], Bhabani Shankar Das[1,#], Lipsa Leena Panigrahi[1], Manoranjan Arakha[1,*] and
and Debapriya Bhattacharya[1,2,*]

[1]*Centre for Biotechnology, Siksha 'O' Anusandhan (Deemed to be University), Bhubaneswar, Odisha, 751003, India;*
[2]*Department of Biological Sciences, Indian Institute of Science Education and Research (IISER) Bhopal, Bhopal, India*

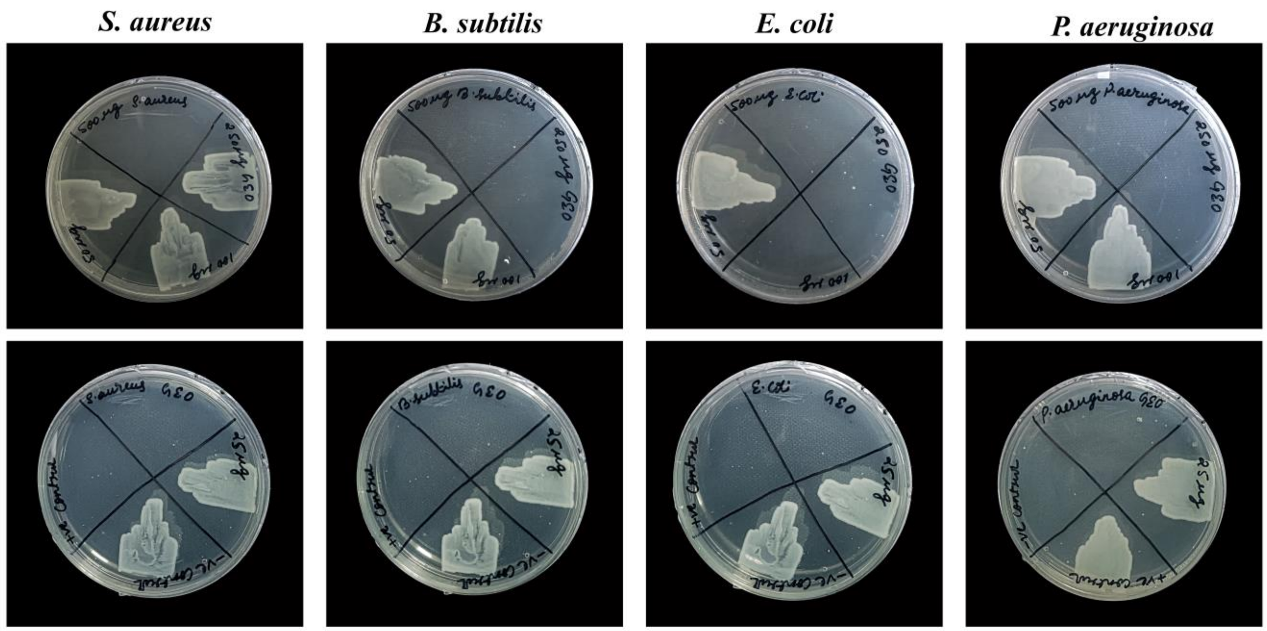

Supplementary Fig. (1). Minimum bactericidal concentration (MBC) of GEO against tested bacteria. (a) *S. aureus* (500 µg/mL); (b) *B. subtilis* (250 µg/mL); (c) *E. coli* (100 µg/mL); (d) *P. aeruginosa* (250 µg/mL) The test was carried out in triplicates.

1873-4294/24 © 2024 Bentham Science Publishers

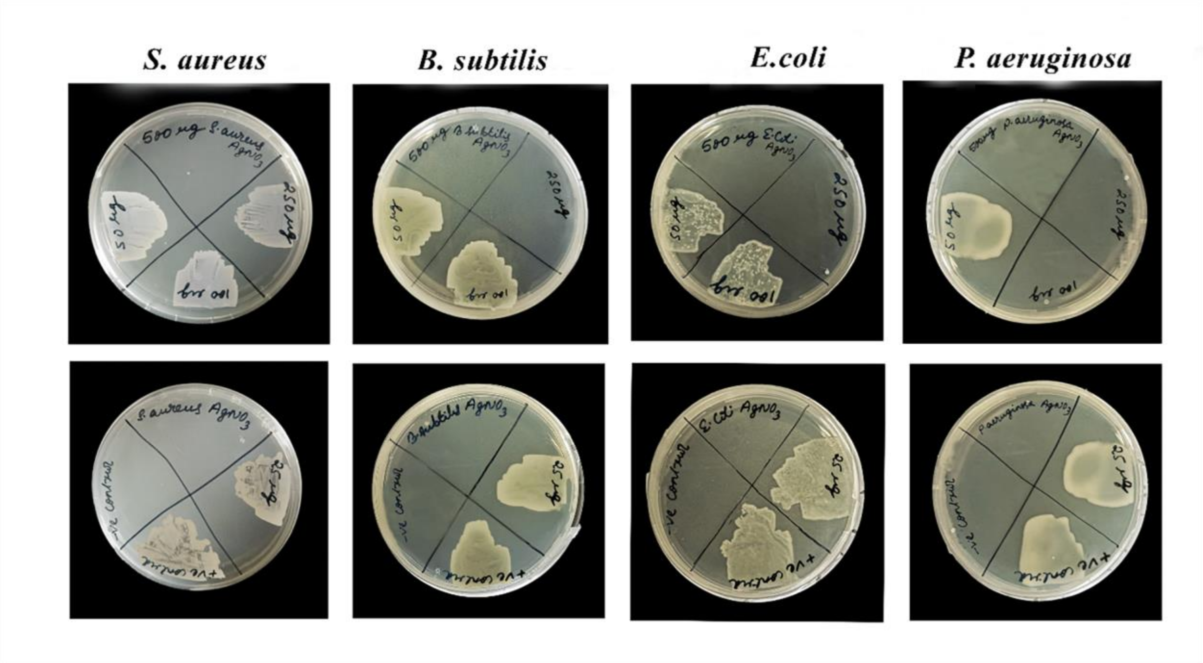

Supplementary Fig. (2). Minimum bactericidal concentration (MBC) of silver nitrate against tested bacteria. (a) *S. aureus* (500 µg/mL); (b) *B. subtilis* (250 µg/mL); (c) *E. coli* (250 µg/mL); (d) *P. aeruginosa* (100 µg/mL) The test was carried out in triplicates.

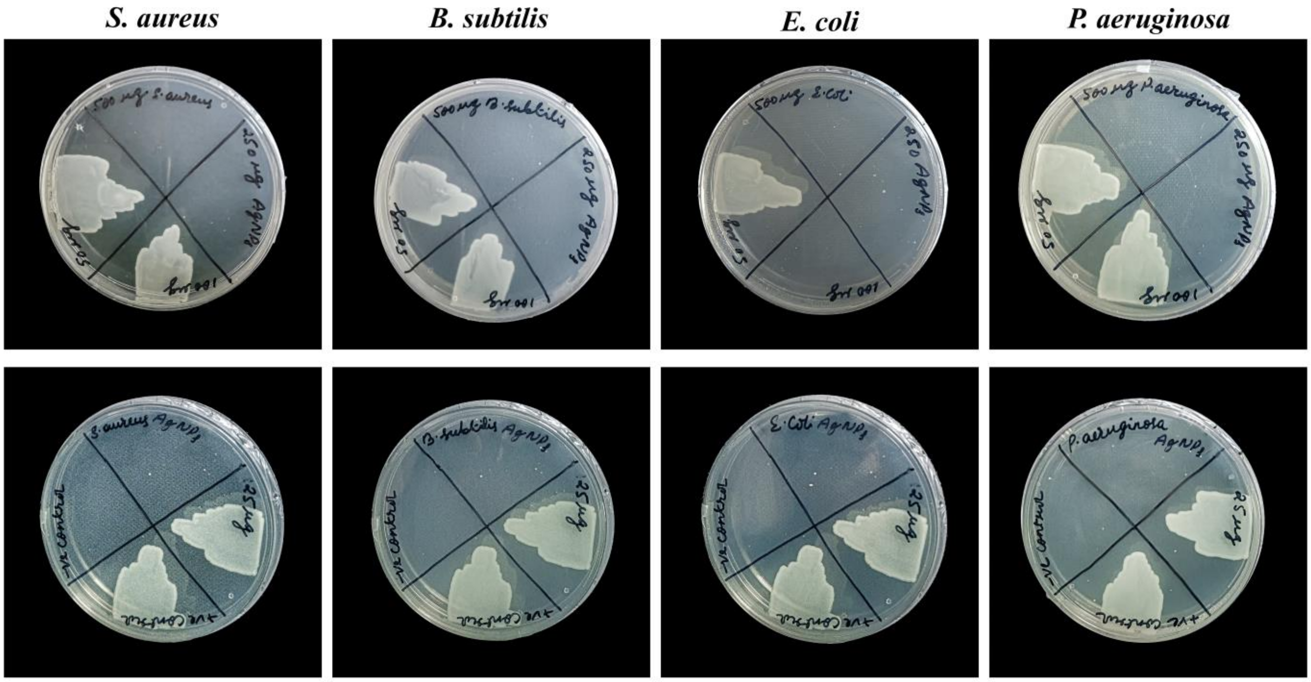

Supplementary Fig. (3). Minimum bactericidal concentration (MBC) of AgNPs against tested bacteria. (a) *S. aureus* (250 µg/mL); (b) *B. subtilis* (250 µg/mL); (c) *E. coli* (100 µg/mL); (d) *P. aeruginosa* (250 µg/mL) The test was carried out in triplicates.

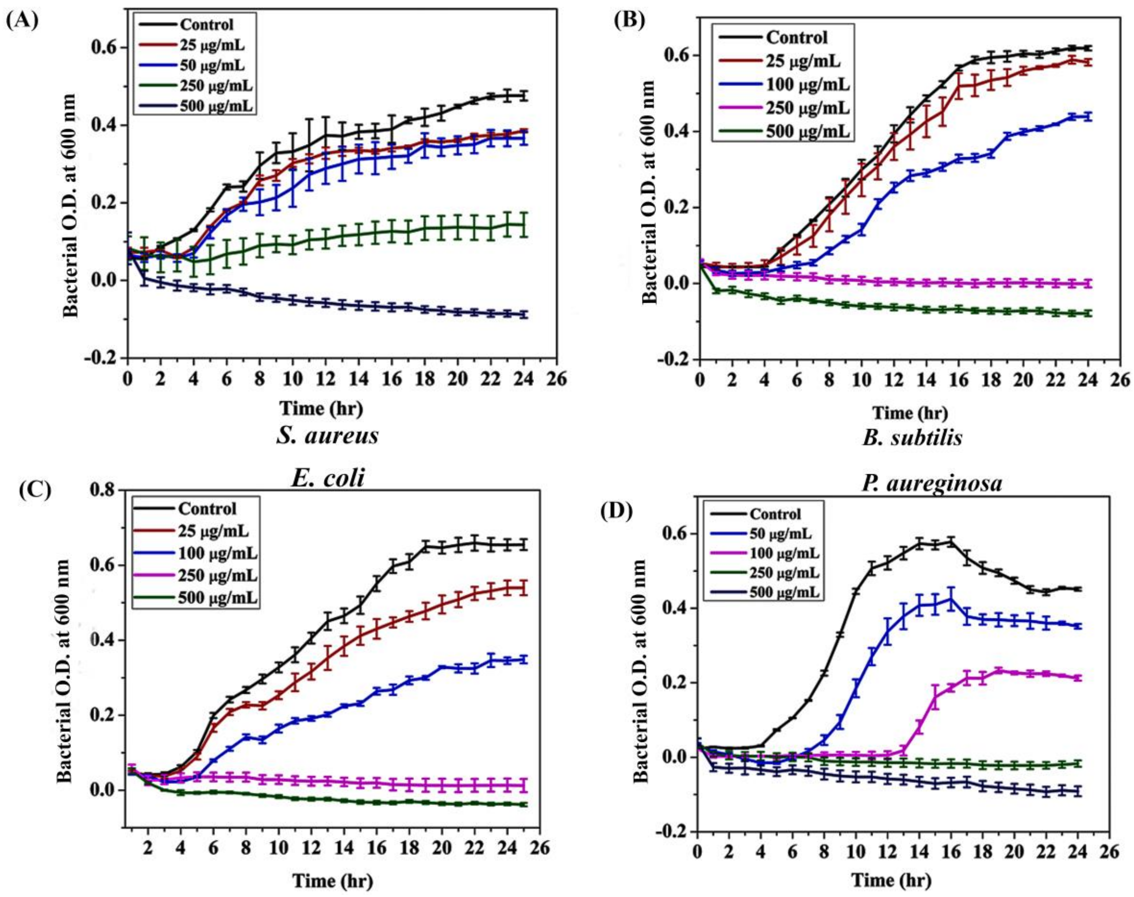

Supplementary Fig. (4). Illustrates dose dependent growth inhibitory effect of AgNPs in all tested strains (A) *S. aureus* (B) *B. subtilis* (C) *E. coli* (D) *P. aeruginosa*. The resulted data are presented as mean ± SD of three separate experiments.

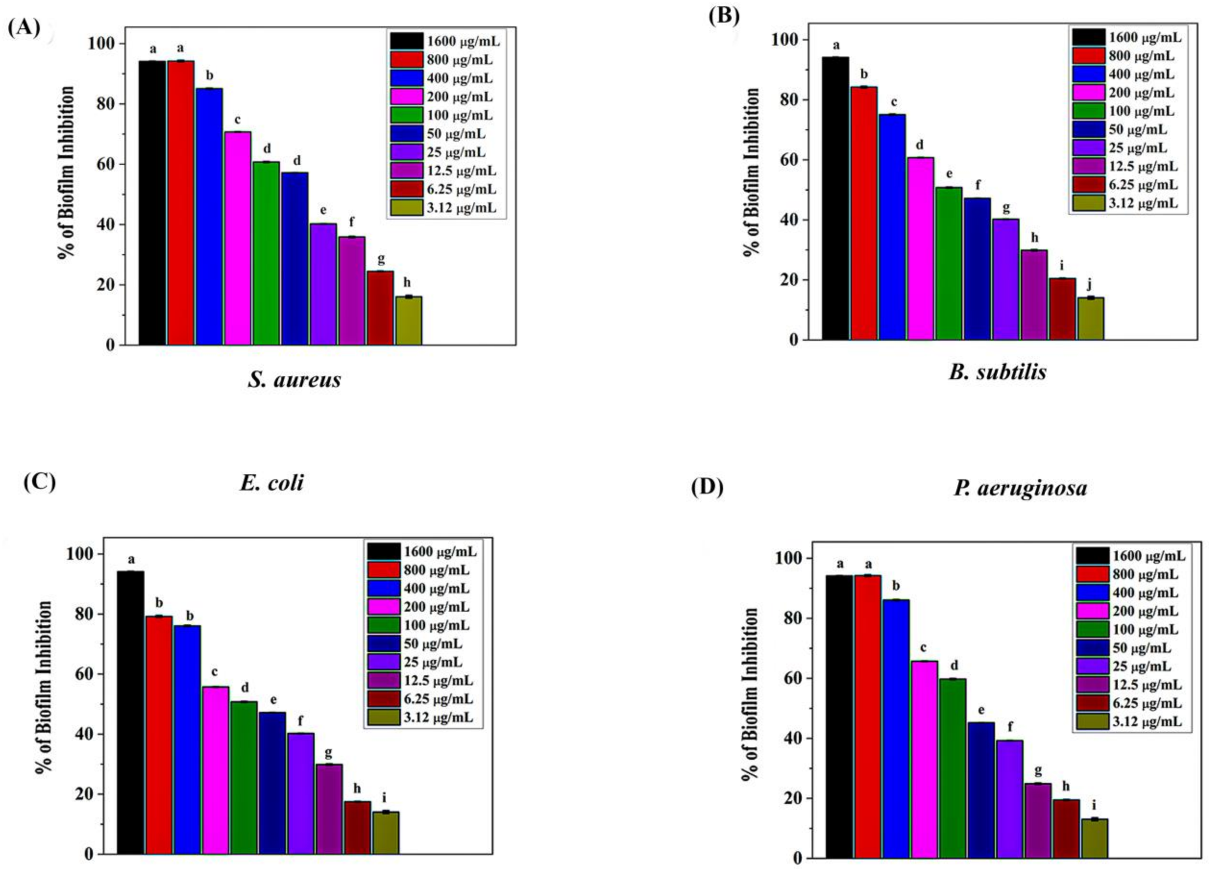

Supplementary Fig. (5). Antibiofilm activity of GEO against biofilm forming tested microorganisms: (A) *S. aureus* (B) *B. subtilis* (C) *E. coli* (D) *P. aeruginosa.* The results are shown as mean ± SD of three individual biological experiments. Individual letters represent significant different compared to the control group (without GEO) with p-value ≤ 0.05.

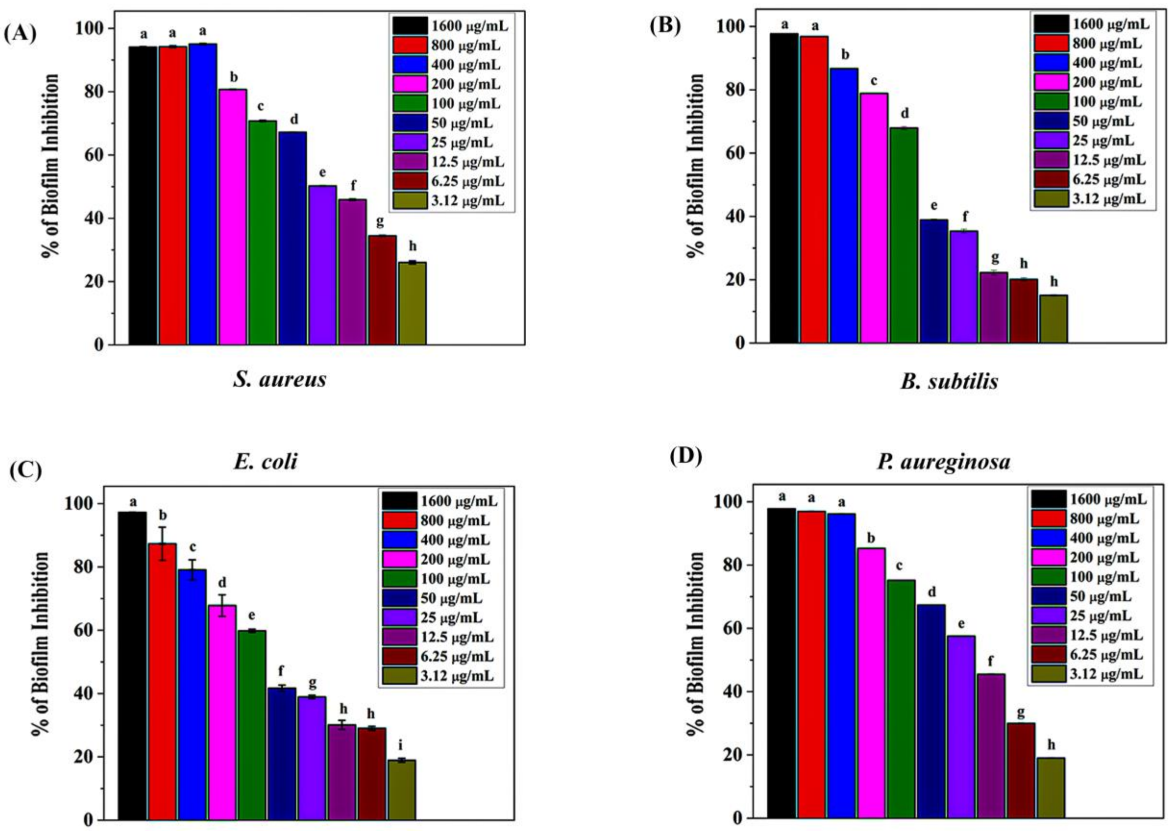

Supplementary Fig. (6). Antibiofilm activity of $AgNO_3$ against biofilm forming tested microorganisms: (A) *S. aureus* (B) *B. subtilis* (C) *E. coli* (D) *P. aeruginosa.* The results are shown as mean ± SD of three individual biological experiments. Individual letters represent significant different compared to the control group (without silver nitrate) with p-value $\leq$ 0.05.

DISCLAIMER: The above article has been published, as is, ahead-of-print, to provide early visibility but is not the final version. Major publication processes like copyediting, proofing, typesetting and further review are still to be done and may lead to changes in the final published version, if it is eventually published. All legal disclaimers that apply to the final published article also apply to this ahead-of-print version.
